# A Paradigm Shift in SSTI Management: The Multifunctional Role of Extracellular Vesicles

**DOI:** 10.3390/ijms26136481

**Published:** 2025-07-05

**Authors:** Barathan Muttiah, Alfizah Hanafiah

**Affiliations:** 1 Department of Medical Microbiology and Immunology, Faculty of Medicine, Universiti Kebangsaan Malaysia, Cheras, Kuala Lumpur 56000, Malaysia; 2 GUT Research Group, Universiti Kebangsaan Malaysia, Cheras, Kuala Lumpur, 56000, Malaysia

**Keywords:** extracellular vesicles (EVs), skin and soft tissue infections (SSTIs), mesenchymal stem cells (MSCs), antimicrobial resistance, wound healing

## Abstract

Skin and soft tissue infections (SSTIs) are becoming an urgent public health issue worldwide. The globe is facing a growing problem with drug-resistant germs, and current treatments are not quite cutting it. There is a real need for new therapies that can tackle these challenges more effectively. This brings us to an interesting question: Can extracellular vesicles (EVs) from different sources, such as mesenchymal stem cells (MSCs), immune cells, or even plants and animals, help in treating SSTIs, especially given the rise in drug resistance? Studies have shown that MSC-derived EVs are particularly noteworthy because they carry components such as antimicrobial peptides (AMPs) that can work together to fight infections, boost the immune response, and aid in healing. These vesicles play a role in how our body interacts with infections, helping to clear bacteria, reduce inflammation, and promote tissue repair. We also see that EVs from plants and bacteria can directly fight off germs, while those from animals can support the healing process of skin. Although early studies have shown promise for EV therapies, there are still hurdles to overcome, such as ensuring consistent production and delivery. This review looks at the potential of EVs as powerful agents in managing infections and supporting healing, highlighting an exciting area of research in medicine.

## 1. Introduction

Skin and soft tissue infections (SSTIs) are a diverse group of bacterial infections of the skin, subcutaneous tissue, fascia, and muscle planes [1]. They range from the mildest presentations (cellulitis) to more serious ones (necrotizing fasciitis) and are among the most common infections both in the community and in the hospital setting [2]. SSTIs are particularly prevalent among individuals with diabetes, peripheral vascular disease, or compromised immunity [3,4] and are the third most frequent cause of emergency department visits [3].

Globally, SSTIs have an estimated rate of 24.6 per 1000 person-years, with higher rates found in high-income nations such as the U.S. (77.5 per 1000 person-years) [5]. Hospitalization increased by 29% in the early 2000s, most frequently because of community-associated methicillin-resistant *Staphylococcus aureus* CA-MRSA [6,7]. Risk is age-, sex-, and socioeconomic-status-dependent, with highest incidence rates in the elderly and high-risk groups [5,6,7]. Hospital data estimate SSTIs contribute 7–10% to infection load [7], with unacceptably high incidences among diabetic patients—up to 86.5% in a study in Pakistan [8]. Resistance is also common, found in 47% of SSTIs in a Ugandan study on multidrug-resistant organisms [9] and over 64% in an Indian tertiary care hospital on *Enterococcus faecium*, *Staphylococcus aureus*, *Klebsiella pneumoniae*, *Acinetobacter baumannii*, *Pseudomonas aeruginosa*, and *Enterobacter* (ESKAPE) pathogens [10].

Despite the fact that 70–75% of SSTI patients are treated in outpatient care, recurrence, antibiotic resistance, and failure of therapy impose a significant health burden [11]. Methicillin-resistant *Staphylococcus aureus* (MRSA) remains a heavy burden, with prevalence of SSTI-associated in Asia ranging from 7.3% to 74% and similarly high elsewhere [12,13]. The emergence of community-associated MRSA (CA-MRSA) has also made things challenging in both hospitals and communities [14]. SSTIs are more prevalent among men (60–70%) and occur most frequently in the 45–64-year age group [15], although rising hospitalization rates in younger adults indicate changing risk factors [16].

**Figure 1:**
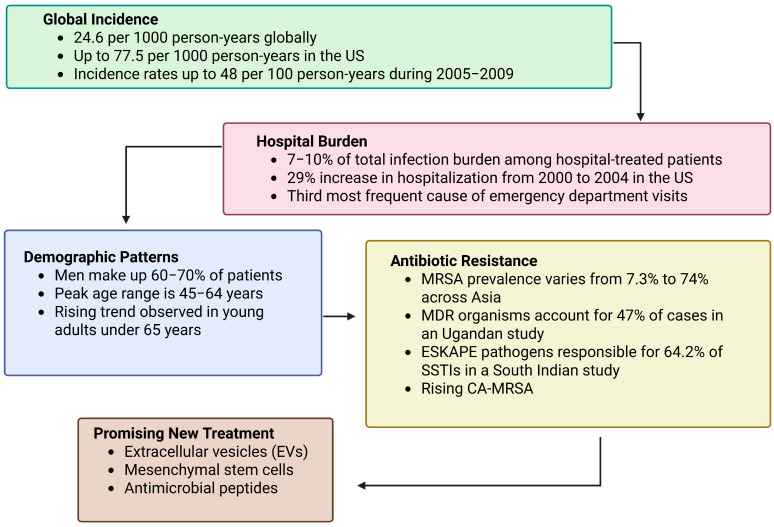
Overview of the epidemiological burden, risk factors, and emerging therapies for SSTIs.

In the face of mounting drug resistance and limitations of current treatments, novel approaches are gaining traction. Mesenchymal stem cells (MSCs) and their secretome of antimicrobial peptides and extracellular vesicles (EVs) have demonstrated promise with immunomodulatory and antimicrobial functions [17,18], notably against such pathogens as *S. aureus*, *Streptococcus pyogenes*, and *Clostridium perfringens* [19]. EVs from MSCs, immune cells, bacteria, plants, and animals have bioactive cargo (miRNAs, proteins, lipids) to regulate inflammation, enhance tissue repair, and combat infection. Their direct application in SSTI treatment is yet to be fully investigated. This mini-review identifies the therapeutic use of MSC-derived and other biologically derived EVs for SSTI therapy, with emphasis on mechanisms of action, current evidence, and translational potential against antibiotic resistance. Figure 1 depicts global incidence trends, hospital burden, risk populations, and imperatives of antibiotic resistance and highlights emerging treatment platforms such as MSCs, EVs, and AMPs.

## 2. Key Bacterial Pathogens in SSTIs

SSTIs represent a broad spectrum of clinical syndromes and are caused by a wide variety of bacterial pathogens. Of these, the most frequently implicated are *S. aureus* and *S. pyogenes* (Group A Streptococcus, GAS), although the offending pathogen will generally differ based on infection, exposure, and host factors such as immune status and comorbid conditions.

### 2.1. Streptococcus aureus

*S. aureus* is the predominant and most common pathogen in SSTIs, presenting with conditions ranging from impetigo to necrotizing fasciitis and cellulitis [20,21]. Its ability to colonize the skin and mucosa, along with the predispositions offered by such conditions as diabetes or immunosuppression, may increase its occurrence. Of particular concern is methicillin-resistant *S. aureus* or MRSA, carrying the mecA gene that encodes PBP2a, which then confers beta-lactam resistance [22]. MRSA causes higher morbidities, treatment failures, and healthcare costs both in-community (CA-MRSA) and in-hospital (HA-MRSA) [23,24]. Treatment often consists of prolonged courses of antibiotics, and IV therapy and drug monitoring may be required in case of invasive infections [24]. Both MRSA and methicillin-sensitive *S. aureus* express numerous virulence factors, including adhesions proteins (fibronectin-binding proteins, EbpS, ClfA/B), the ability to form biofilms, immune evasion mechanisms (Protein A and capsular polysaccharides), and tissue-destructive toxins (α-toxin) [25,26,27,28,29,30,31]. CA-MRSA strains, such as USA300, often carry Panton–Valentine leukocidin (PVL), linked to tissue necrosis, and show hyperactive agr quorum-sensing systems that enhance toxin production [32,33,34]. Additional virulence and resistance genes carried on mobile genetic elements such as staphylococcal cassette chromosome mec (SCCmec) and arginine catabolic mobile element (ACME), aside from causing antibiotic resistance, enhance bacterial colonization, persistence, and virulence [35]. Wall teichoic acids and, in some strains, TSST-1 further amplify the pathogenicity of MRSA [36,37]. While MRSA is more often linked with adverse outcomes, some MSSA strains, such as ST398, are also highly virulent [38]. Clinical studies suggest higher morbidity and mortality with MRSA due to resistance and virulence synergy, though experimental data on relative virulence remains mixed, with strain variability and host factors playing significant roles [39,40,41]. Empirical treatment often includes clindamycin, doxycycline, TMP-SMX, or vancomycin depending on resistance and severity.

### 2.2. Group A Streptococcus (Streptococcus pyogenes)

*Streptococcus pyogenes* is a key cause of SSTIs, particularly cellulitis and erysipelas. Cellulitis affects deeper, subcutaneous tissues, while erysipelas is more superficial, involving the upper dermis and lymphatics and leading to severe conditions such as necrotizing fasciitis and streptococcal toxic shock syndrome (STSS) [42,43]. Both present with redness, heat, and swelling, and erysipelas especially shows raised, well-defined borders. It can progress rapidly and often involves lymphatic streaking [42]. Meanwhile, noninvasive infections such as pharyngitis, impetigo, and scarlet fever are typically localized and transmitted via respiratory droplets or direct contact [43]. Postinfectious sequelae, including rheumatic fever and APSGN, arise from autoimmune or immune complex-mediated responses occurring weeks after the initial infection [44]. *S. pyogenes* exhibits a wide range of clinical manifestations categorized by infection type and serotype. Over 80 M protein serotypes determine strain-specific virulence, with Class I strains linked to rheumatic fever and Class II strains associated with acute poststreptococcal glomerulonephritis (APSGN) [45,46]. The M protein is a key virulence factor that aids in immune evasion by inhibiting phagocytosis and forms the basis for strain typing [47]. It invades tissues rapidly through various virulence factors, including M protein, which inhibits phagocytosis; streptolysins and hyaluronidase, which promote tissue degradation; and superantigens, which trigger excessive immune activation [48]. Other key virulence factors include Streptolysin O (SLO), an oxygen-labile, immunogenic cytotoxin that forms pores in host cell membranes, causing lysis of erythrocytes, leukocytes, and platelets. It also induces production of anti-streptolysin O antibodies (ASO), used diagnostically. Meanwhile, streptolysin S (SLS) is an oxygen-stable, nonimmunogenic cytotoxin responsible for beta-hemolysis on blood agar and cytotoxic to host cells [49]. Pyrogenic exotoxins (SpeA, SpeB, SpeC) are superantigens that induce cytokine storms in diseases such as scarlet fever and streptococcal toxic shock syndrome; SpeB also has a role as a protease that breaks down host tissue [50]. Furthermore, streptokinase converts plasminogen to plasmin, which promotes fibrin degradation and bacterial spread [51]. Hyaluronidase and DNases also facilitate tissue invasion and NET evasion by degrading connective tissue and extracellular DNA, respectively [52]. Protein F and lipoteichoic acid enhance adherence to epithelial cells, enabling colonization, and C5a peptidase prevents neutrophil recruitment by degrading C5a [53]. Additional proteases, amylases, and esterases are involved in immune modulation, tissue destruction, and systemic dissemination, making *S. pyogenes* a highly versatile and invasive pathogen [54]. Treatment of severe infections involves high-dose intravenous penicillin in combination with clindamycin to inhibit bacterial protein synthesis and reduce toxin production.

### 2.3. Clostridium perfringens

*Clostridium perfringens* is an anaerobic, spore-forming, Gram-positive bacillus that plays a key role in the pathogenesis of life-threatening soft tissue infection, primarily gas gangrene (clostridial myonecrosis) [55]. The bacterium frequently infects contaminated deep wounds, as in traumatic injury, crush injury, and surgical wounds, in which anaerobic conditions promote bacterial multiplication and spore germination [56]. The harm caused by *C. perfringens* is largely attributed to its powerful exotoxins, especially alpha-toxin (lecithinase), which disrupts cell membranes through phospholipid hydrolysis, causing extensive tissue necrosis, hemolysis, and vascular leakage [57]. Another major toxin, theta-toxin (perfringolysin O), forms pores in host cell membranes and causes vascular damage, myocardial depression, and circulatory collapse, contributing to systemic toxicity [58]. Treatment requires prompt and aggressive surgical debridement to remove necrotic tissue, along with high-dose intravenous penicillin to suppress bacterial growth [59]. Hyperbaric oxygen therapy is often employed as an adjunct therapy, utilizing the oxygen-dependent metabolism of anaerobic bacteria to inhibit growth and enhance healing [60]. Early diagnosis and timely intervention are essential to maximizing results in clostridial infection patients.

### 2.4. Others

While *S. aureus* and GAS dominate the SSTI realm, there are other pathogens with significant roles under specific clinical conditions. Gram-negative bacilli such as *Pseudomonas aeruginosa*, *Escherichia coli*, and *Aeromonas hydrophila* are significant pathogens of SSTIs after exposure to freshwater. *P. aeruginosa* is endemic in water sources such as pools and hot tubs and can lead to folliculitis, wound infections, and severe burn wound infections with blue-green discharge and drug resistance due to biofilm production [61]. *E. coli*, especially pathogenic species such as O157:H7, can have the potential to cause SSTIs in contaminated freshwater systems, though more notoriously for gastrointestinal infection [62]. *A. hydrophila*, which is a common inhabitant of freshwater and brackish environments, is a causative agent of a spectrum of SSTIs, from cellulitis to necrotizing fasciitis, predominantly in water-exposed wounds. It secretes tissue-degrading enzymes and possesses increasing multidrug resistance and is thus an emerging pathogen in burn and trauma infections [63].

Pathogens of animal bites such as *Pasteurella multocida* (most commonly from cats), *Pasteurella canis* and *Capnocytophaga canimorsus* (from dogs), and *Eikenella corrodens* (from human bites) can potentially produce rapidly progressing infections that extend from localized injuries to full-blown systemic disease [64]. *C. canimorsus*, a Gram-negative rod found in the oral flora of dogs and cats, typically produces symptoms 3 to 5 days after a bite or scratch [65]. Infection can promptly progress from nonsignificant local symptoms (swelling, erythema, blisters, pain, purulence) to systemic infection, sepsis, and potentially death, most notably in hosts who are immunocompromised such as asplenic individuals or those who have liver disease [66]. It may cause fulminant septicemia with shock, disseminated intravascular coagulation, and multiple organ failure. Meningitis and severe sepsis are also seen in immunocompetent individuals [67]. Prompt wound cleansing in the initial stages and prompt antibiotic treatment (penicillin G or beta-lactam/beta-lactamase inhibitor combinations) are key to prevention. *Pasteurella multocida*, transmitted primarily through cat bites, is infamous for inducing spreading soft tissue infections with swelling, erythema, and pain within 24 h of the bite. It can produce cellulitis, abscess, and systemic infection if not treated [68]. *Pasteurella canis* and *Capnocytophaga canimorsus* in dog bites also cause rapidly progressing infections with the potential for systemic dissemination [69]. *E. corrodens*, a human oral flora organism, is implicated in human bite wounds and clenched fist injury. It causes virulent soft tissue infections that can progress rapidly without intervention [70].

*Vibrio vulnificus* and *Mycobacterium marinum* are notable sea- and freshwater-borne pathogens that cause serious SSTIs, mostly in immunocompromised individuals [71,72]. *V. vulnificus*, a bacterium found in warm seawater, causes progressing wound infections and necrotizing fasciitis, typically acquired by exposure of an open wound or raw seafood ingestion [71]. It is highly fatal and requires immediate antibiotic treatment and surgery. *M. marinum*, present in aquarium water, causes chronic localized infection such as “fish tank granuloma” following trivial trauma to the skin [72]. It requires a prolonged course of antibiotic treatment and sometimes surgery. Prevention is by avoiding contact with infected water and wound care [73]. Table 1 shows the major bacterial pathogens responsible for SSTIs, including the common types of infections they cause, their principal virulence factors, known resistance mechanisms or notable strains, and key treatment considerations.

## 3. Epidemiological Evidence of Antibiotic Resistance in SSTIs

Emergence of antimicrobial resistance in SSTIs is becoming a global concern with regional and organism-specific variations. MRSA is the major resistant pathogen, accounting for 44% of SSTIs in Uganda [74] and having risen in Gabon from 3% over an 11-year period to 20% [75]. MRSA isolates were 94% resistant in Taiwan towards erythromycin and clindamycin but susceptible towards linezolid, teicoplanin, and vancomycin [76]. While in Europe the prevalence of MRSA is below 1% in some countries [77], in some parts of South America, Asia and the US, it is more than 60%, and it may be greater than 50% in some African regions [78]. In Malaysia, MRSA constituted 19.4% of blood isolates of *S. aureus* from 41 hospitals [79]. In US emergency-department-related SSTI cases, MRSA accounted for 54.8% of cases [80], whereas 4.5% of shelter residents were found to be the 2006 prevalence [81].

Resistance among Group A Streptococcus (GAS) to macrolides and lincosamides is also increasing, with erythromycin resistance reported as high as 90% and clindamycin resistance up to 50% in some regions [82,83]. This geographic variability hinders empirical therapy, particularly for those allergic to penicillin [84]. A study conducted in Kuala Lumpur reported 42.1% of surgical site infection isolates as MDROs [85]. Clostridium perfringens shows resistance to multiple drugs including tetracycline, lincomycin, erythromycin, clindamycin, and cefotaxime [86]. Additionally, 61% of Gram-negative SSTI isolates were ESBL producers [87], and 27% showed carbapenem resistance [88]. Pseudomonas aeruginosa remains challenging due to intrinsic and acquired resistance [89], while multidrug-resistant *Aeromonas hydrophila* strains have been found in both clinical and environmental settings, suggesting possible transmission routes [90]. These trends underscore the need for continuous surveillance, tailored empirical treatment, infection control, and antimicrobial stewardship to address the rising threat of resistance in SSTIs.

## 4. Current Treatment for SSTIs

Accurate diagnosis, early treatment, and stratified management based on the type and severity of the infection are the cornerstones for the treatment of SSTIs. Diagnosis is mainly clinical, based on signs such as erythema, edema, pain, pus drainage, and systemic signs of fever, which suggest a serious infection [91]. Wound cultures should be performed so as to steer antibiotic therapy. A 5-day course of antibiotic therapy is usually indicated for simple infections and may be extended if the patient is slow to respond [92]. Supportive care such as limb elevation, wound care, and hygiene education must also be given [93]. Other than abscesses, purulent SSTIs are usually treated with incision and drainage, while reserving antibiotics for patients presenting systemic symptoms, or those with immunosuppression or in cases of treatment failure [94]. Empiric oral options include TMP-SMX, doxycycline, or clindamycin; IV options include vancomycin or linezolid. Nonpurulent SSTIs (cellulitis) are mostly caused by streptococci and MSSA and are treated preferably with cephalexin or IV cefazolin; vancomycin is used if an MRSA infection is suspected or if there are allergies against beta-lactams [95]. Necrotizing fasciitis is a surgical emergency in which immediate debridement should be performed along with the institution of empiric IV broad-spectrum antibiotics (e.g., vancomycin plus cefepime, or piperacillin-tazobactam) [96]. Clindamycin or linezolid may also be given for toxin suppression [97]. Animal bites are usually treated with amoxicillin/clavulanate, or doxycycline for those allergic to penicillin. Mild folliculitis can be managed with topical therapies, whereas extensive folliculitis might require systemic treatment [98]. Recurrence can be reduced by follow-up in 48 to 72 h, adjusting antibiotics as guided by culture, and addressing risk factors [99].

With recent treatment advances, outcomes have improved, especially in resistant, toxin-producing, and polymicrobial infections. Newer agents such as delafloxacin and omadacycline span broad coverage, including MRSA and some ESBL-producers [100]. In addition, long-acting lipoglycopeptides, namely dalbavancin and oritavancin, allow for a once-weekly dosing schedule, rendering these drugs potentially viable candidates for outpatient treatment of SSTIs [101]; however, their exact role in the management of severe SSTIs still needs to be well elucidated. Clindamycin, linezolid, and tedizolid (a next-generation oxazolidinone with less side effects) remain important for toxin suppression in TS [102,103]. Empiric combination regimens, especially a combination of a broad-spectrum β-lactam (piperacillin–tazobactam or cefepime) with a toxin-suppression agent such as clindamycin or linezolid, are increasingly being employed for necrotizing SSTIs and have shown improved outcomes [104].

Adjunctive therapies such as IVIG may possibly work against streptococcal toxic shock by neutralizing superantigens [105], whereas HBOT could be beneficial for necrotizing infections but should never delay definitive treatment [106]. Treatment options now focus prominently on PK/PD-based antibiotic dosing to maximize penetration into tissues with poor perfusion, with prolonged or continuous infusion of β-lactams and serum-level monitoring of drugs such as vancomycin being a good example in achieving high efficacy and low toxicity [107,108]. In parallel, regenerative strategies include the use of MSCs and their EVs. MSCs carry out immunomodulatory and tissue-repair functions, mainly being useful in chronic or ischemic SSTIs. In addition, MSC-derived extracellular vesicles deliver miRNAs, cytokines, and growth factors to injured tissues, which favor repair processes sans any risks connected with live cell therapies. Although this is mostly proof-of-concept and preclinical, these early translational studies imply that MSCs and EVs could become adjunctive therapies for complex drug-resistant SSTIs in the near future. Table 2 summarizes the clinical management strategies for various types of SSTIs, including first-line antimicrobial treatments, adjunctive therapies, and recent advances in pharmacological and regenerative approaches.

## 5. Extracellular Vesicles (EVs)

EVs are cell-secreted, membrane-bound nanoparticles from nearly all cell types. Based on their cell origin, size, and biogenesis, EVs are broadly classified into small EVs (sEVs) and large EVs (lEVs) [109]. An understanding of how they are generated and how they choose their cargo is essential in determining their physiological and pathological functions, e.g., immunity, infection, and oncogenesis [110]. Exosomes or sEVs are small vesicles measuring 30–150 nm derived from the endosomal pathway. Exosome biogenesis begins with inward budding of the early endosomal limiting membrane, which creates MVBs that can either fuse with lysosomes to break down the cargo or fuse with the plasma membrane and expel intraluminal vesicles (ILVs) as exosomes into the exterior space [111,112]. Exosome formation occurs by two main routes: the ESCRT-dependent mechanism, where ESCRT-0, -I, -II, and -III complexes and core components such as TSG101 (ESCRT-I) and ALIX (ESCRT-III accessory protein) act sequentially to direct membrane budding and sorting of cargo; and the ESCRT-independent mechanism, which relies upon lipid raft domains and tetraspanin-enriched microdomains (TEMs), where tetraspanins including CD9, CD63, and CD81 facilitate vesicle formation and cargo selection [113]. Regulatory proteins such as Rab GTPases (e.g., RAB27a/b, RAB11) regulate MVB trafficking and docking, whereas membrane fusion is regulated by SNARE proteins to allow exosome release [114]. The endoplasmic reticulum (ER) is also involved in the membrane dynamics and maturation of MVB. Exosomes are often identified by some molecular markers such as tetraspanins (CD9, CD63, CD81), ESCRT-related proteins (ALIX, TSG101), and other markers such as flotillin, HSP70, and HSP90 [115,116].

**Figure 2:**
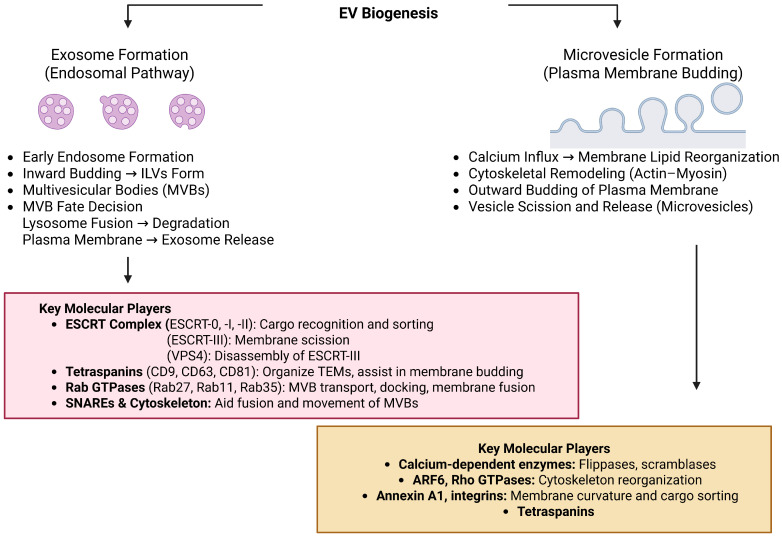
The biogenesis and release of EVs. Abbreviations: EVs (Extracellular Vesicles); ILVs (Intraluminal Vesicles); ESCRT (Endosomal Sorting Complex Required for Transport); VPS4 (Vacuolar Protein Sorting-Associated Protein 4); CD9/CD63/CD81 (Tetraspanins—common EV surface markers); TEMs (Tetraspanin-Enriched Microdomains); Rab GTPases (Ras-related GTP-binding proteins involved in vesicle trafficking); Rab27/Rab11/Rab35 (Rab family GTPases regulating EV secretion); SNARE (Soluble NSF Attachment Protein Receptor); ARF6 (ADP-Ribosylation Factor 6); Rho GTPases (Ras Homolog GTPases involved in cytoskeletal dynamics); Annexin A1 (Phospholipid-binding protein involved in EV biogenesis and trafficking).

lEVs, otherwise referred to as microvesicles or ectosomes, measure approximately 100 to 1000 nm in diameter, but some subpopulations such as large oncosomes measure more than 1 µm in diameter [117]. In contrast to small EVs (exosomes) of the endosomal route, lEVs are created through direct plasma membrane outward budding, and fission and MVBs do not play a role in their biogenesis. lEV production is typically evoked by a rise in intracellular calcium leading to cell membrane phospholipid asymmetry disruption and activation of flippases, floppases, and scramblases to induce membrane curvature [118]. Concurrently, calcium entry facilitates cytoskeleton remodeling via actin filament reorganization that enhances cell membrane protrusion and vesicle shedding. Small GTPases such as ARF6 and members of the Rho family are implicated in membrane traffic, reorganization of the cytoskeleton, and selective cargo loading [119]. Microvesicle formation is generally not dependent on ESCRT machinery, although some ESCRT components may be implicated under specific conditions. Molecular lEV markers include Annexin A1, which in a calcium-dependent manner is a phospholipid binder and contributes to membrane curvature; ARF6, which controls cytoskeletal dynamics; and integrins, particularly in microvesicles from invasive or migrating cells [120]. The heterogenous lEV cargo is typically enriched with ribosomal proteins, factors involved in RNA processing, cytosolic enzymes, and signaling molecules and associated with inflammation, coagulation, cellular stress response, and cancer growth [121]. Table 3 shows the comparison of different types of EVs. Figure 2 shows the biogenesis and release of EVs.

### 5.1. EVs in Promoting SSTIs

In SSTIs, EVs are archetypal characters involved in infection dynamics, immune modulation, and inflammation of tissue. They carry a heterogeneous cargo of proteins, lipids, nucleic acids, and pathogen-associated molecular patterns (PAMPs), which enable them to affect both host defense mechanisms and pathogen virulence processes [122]. In bacterial SSTIs, *S. aureus* releases cytoplasmic membrane vesicles (CMVs), also known as *S. aureus*-derived EVs (SA-EVs), and are loaded with virulence factors such as α-hemolysin, proteases, β-lactamases, and nucleic acids. These vesicles can traverse the epidermal barrier, activate keratinocytes, and initiate inflammatory cascades by cytokines such as IL-6 and TNF-α, compromising the integrity of the skin and exacerbating infections such as impetigo and atopic dermatitis (AD) [123]. SA-EV secretion is induced in response to environmental stressors such as ultraviolet (UV) light and certain antibiotics, enhancing infection severity [124,125]. SA-EVs also contribute to AD by promoting antibiotic resistance through beta-lactamase delivery, disrupting the epidermal barrier via alpha-hemolysin and protein A, and inducing inflammation [126]. CMVs also mediate antibiotic resistance by transferring β-lactamases, disrupt the epidermal barrier via α-hemolysin and protein A, and promote inflammatory responses [127]. CMVs are also capable of regulating immune responses by inducing proinflammatory cytokines (CXCL8, TNF-α), neutrophil recruitment, and immune cell infiltration, both promoting pathogen clearance and tissue damage [128]. CMVs also enable intercellular communication between *S. aureus* populations to enable coordinated expression of the resistance and virulence genes [129].

SA-EVs are also responsible for biofilm formation through enhanced hydrophilicity of the skin surface, enhanced bacterial colonization, and shielding of the pathogen against host immune systems. This biofilm-forming activity is the cause of chronic and recurring infections [130]. In chronic wounds, particularly polymicrobial biofilms such as diabetic foot ulcers, EVs from *S. aureus* and *P. aeruginosa* deliver quorum-sensing molecules and extracellular matrix components that reinforce biofilm maturation, modulate immune responses, and promote antibiotic resistance [131]. These mechanisms collectively delay wound healing and perpetuate infection chronicity, highlighting the critical role of bacterial EVs in SSTI pathogenesis and persistence. Membrane vesicles (MVs) carry proteins such as Staphylococcal protein A (SpA), which binds host IgG and blocks complement activation, helping bacteria evade immune clearance. They also induce proinflammatory cytokines via activation of innate immune receptors (TLR2), which can paradoxically contribute to tissue damage and facilitate bacterial spread [132]. The similar MVs also enable the transport of the various factors, such as ferritin and lipoproteins, responsible for iron acquisition. Iron is limited in hosts and is essential for bacterial growth [133].

In addition, Gram-negative bacterial outer membrane vesicles (OMVs) facilitate tissue invasion during infections via several mechanisms based on delivering their virulence factors to host cells, activating host immune responses and aiding in the colonization and dissemination of bacteria [134]. OMVs are filled with numerous virulence factors, including lipopolysaccharide (LPS), outer membrane proteins (OmpA), toxins (Shiga toxin and cholera toxin), enzymes, and small RNAs that are all delivered directly to host cells that can damage tissues, disrupt boundaries, and allow the bacteria to potentially invade deeper tissues without direct contact with the host cells [135]. OMVs from pathogens such as *Helicobacter pylori* and *Porphyromonas gingivalis* can both degrade mucin layer and tight junction proteins in epithelial cells, disrupting the physical barrier to promote colonization and invasion of tissues [136]. OMV-associated components such as LPS and outer membrane proteins activate pathogenic-associated pattern recognition receptors (TLR4), leading to the activation of signaling pathways (NF-κB, MAPK) that induce, for example, proinflammatory cytokines, promote activation of the inflammasome, and trigger pyroptosis. Inflammation can lead to damage to host tissues, promote vascular permeability, and provide an opportunity for bacteria to spread deep in tissues [137]. OMVs can modulate immune responses to the advantage of bacteria through delivery of factors that may lead to inhibition of immune clearance or even apoptosis of immune cells. An example of this is *Neisseria gonorrhoeae* OMVs that carry PorB protein to macrophage mitochondria, leading to apoptosis and therefore loss of immune function [138]. OMVs also carry factors associated with adhesion (Ail protein in *E. coli*; Ipa proteins in *Shigella flexneri*) and facilitate invasion [139,140]. OMVs can contribute to the formation of the biofilm matrix that can also facilitate persistence in tissues and provide resistance to host defenses and antibiotics [141].

Fungal pathogens, including *Malassezia sympodialis*, *Candida albicans*, and *Cryptococcus neoformans*, also release EVs [142]. For example, EVs from *M. sympodialis* contain allergens such as Mala s1 and s5–13, as well as nucleic acids that promote Th2-type allergic inflammation by inducing IL-4 and ICAM-1 expression in keratinocytes, thereby worsening diseases such as atopic dermatitis [143]. Lysophospholipase in EVs degrades sebaceous lipids, releasing irritating fatty acids that disrupt barrier integrity and increase permeability resulting skin barrier degradation [144]. A study demonstrated that EVs from *Candida albicans* play a pivotal role in the pathogenesis of SSTIs by carrying bioactive molecules, including proteins, lipids, enzymes, and RNAs, which promote biofilm formation, deliver virulence factors, and influence host cell behaviors such as proliferation and collagen deposition, potentially impairing wound healing [145]. Specifically, EVs stimulate cytokine production (IL-6, IL-4, IL-12, IL-10, TGF-β, TNF-α), contributing to local inflammation. During biofilm development, EVs enrich the extracellular matrix, shielding the fungus from antifungal agents and mediating drug resistance. This is evident in ESCRT-deficient mutants, where reduced EV release increases drug susceptibility [146]. *Cryptococcus neoformans*-EVs play a significant role in SSTIs by transferring virulence factors that guarantee fungal survival and host invasion. Vesicles interact with immune cells, particularly macrophages, which alter host responses and exacerbate inflammation. EVs can also guarantee fungal dissemination by impacting the host’s immune signaling. Experiments have shown that EVs impact fungal biology and host–pathogen interaction, resulting in skin lesions in cutaneous cryptococcosis [147]. Table 4 organizes the pathogenic roles of microbial EVs in SSTIs.

### 5.2. EVs in Supressing SSTIs

EVs play a pivotal role in modulating SSTI suppression because they have the dual capability to modulate immune response as well as stimulate tissue regeneration. Released by numerous cell types such as keratinocytes, immune cells, endothelial progenitor cells (EPCs), and MSCs, as well as bacterial, plant, and animal cells, these nanoscale, membrane-bound vesicles harbor bioactive cargos such as proteins, lipids, mRNAs, and microRNAs that allow them to modulate multiple biological processes that are essential for SSTI resolution [148,149].

EVs derived from cells inhabiting the skin such as keratinocytes play a role in innate immune activation by regulating the functions of macrophages, DCs, and neutrophils. For instance, keratinocyte-derived EVs can carry immunoregulatory molecules such as anti-inflammatory cytokines (IL-10, TGF-β), microRNAs (miR-146a, miR-21), and suppressive proteins that modulate the activation state of immune cells. These EVs can interact with macrophages, dendritic cells, and T cells to dampen proinflammatory responses, especially after the resolution of an infection or during wound healing [150]. Keratinocyte-derived EVs also deliver AMPs, such as β-defensin 2 and S100A12, as well as chemoattractants including CXCL1, CXCL3, CXCL5, and CXCL6, which contribute to immune cell recruitment, with broad-spectrum activity and direct antibacterial action on bacteria such as *S. aureus* [151].

Keratinocyte-derived EVs also control local immunity by carrying cytokines and microRNAs that control inflammation, suppressing excessive immune activation while maintaining effective antimicrobial defense. Moreover, KC-EVs enhance skin barrier function by promoting keratinocyte proliferation and migration for re-epithelialization and restoration of barrier integrity after infection-induced tissue damage [152]. Keratinocytes stimulated with *S. aureus* enterotoxin B release EVs containing MHC molecules capable of enhancing CD4^+^ and CD8^+^ T cell proliferation, suggesting an immunostimulatory role in cutaneous defense [153]. A study also demonstrated that keratinocyte EVs have been shown to influence macrophage polarization, promoting a shift from the proinflammatory M1 phenotype to the anti-inflammatory M2 phenotype. M2 macrophages secrete IL-10 and TGF-β, which are critical for tissue repair, extracellular matrix remodeling, and suppression of immune overactivation. This transition is essential for preventing chronic inflammation and facilitating skin regeneration after injury [154]. Keratinocyte-derived EVs contain human β-defensin 3, which influences immune cell behavior, including macrophage polarization and dendritic cell activation. These vesicles not only inhibit microbial growth by directly targeting pathogens but modulate inflammatory signaling to limit tissue damage [155]. Furthermore, keratinocyte EVs facilitate intercellular communication essential for wound healing by promoting keratinocyte proliferation, migration, and angiogenesis. Keratinocyte-EVs are involved in skin homeostasis and wound healing and pigmentation. They carry proteolytic enzymes, such as cathepsin B and matrix metalloproteinase-1, and TGF-binding proteins, which activate fibroblasts to remodel the extracellular matrix and promote keratinocyte migration [156].

Notably, fibroblast-EVs are an extremely heterogeneous cell population, the functional modes of which are regulated by the biological properties of the donor fibroblasts and the microenvironment. This heterogeneity is a reflection of the inherent diversity of fibroblast subtypes in different anatomical locations, and their ability to adapt dynamically to physiological and pathological stimuli, including infection, inflammation, and tissue injury [157]. Fibroblast-EVs enhance keratinocyte migration and protect against oxidative stress, underscoring their role in skin homeostasis and repair. Importantly, fibroblast-EVs from scleroderma patients exhibit dysregulated collagen-related miRNAs that enhance fibrotic responses, suggesting a contribution to disease pathogenesis [158]. For example, in the presence of SSTIs, PAMPs and proinflammatory cytokines can modulate fibroblast activity, leading to the release of EVs with modified cargo that may exacerbate or resolve inflammation. These infection-altered EVs could affect processes such as wound healing, immune cell recruitment, and matrix remodeling, ultimately impacting tissue regeneration and disease progression [159].

Adipocyte-derived EVs have been reported to modulate inflammatory responses by managing cytokine release and the activation of immune cells. Vesicles have been reported to suppress the release of proinflammatory cytokines such as IL-1β, IL-6, and tumor necrosis factor-alpha (TNF-α) and promote the release of anti-inflammatory cytokines such as IL-10. This immunomodulation is required in the process of suppressing collateral tissue damage and promoting a regulated healing response [160]. It can strengthen the host’s resistance to infection-related tissue deterioration. The indirect antimicrobial benefits of restoring vascularization, improving barrier function, and suppressing excessive inflammation are crucial in managing SSTIs and preventing chronic wound formation. Notably, adipose-derived stem cell EVs have been found to inhibit inflammatory mediator production and release, suggesting a therapeutic application in decreasing overactive or chronic inflammation in SSTIs [161] whereby adipose-derived stem cells EVs indeed stimulate ECM remodeling by modulating matrix metalloproteinases (MMPs) and deposition of collagen, critical processes for effective wound healing. These regenerative properties make Ad-EVs good candidates for wound therapy complicated by infection, ischemia, or diabetes [162].

MSC-EVs are a new and hopeful approach to treating SSTIs that offer a cell-free therapeutic agent with a blend of regenerative, immunomodulatory, and antimicrobial functions. MSC-EVs are nanoscale extracellular vesicles secreted by MSCs and are extensively loaded with bioactive molecules, including microRNAs, proteins, and lipids. These vesicles replicate most of MSC’s therapeutic actions but with stability, storage, and safety advantages, thus becoming a potential substitute for live-cell therapies [163]. Perhaps the most significant manner in which MSC-EVs exert their therapeutic activity in SSTIs is through immunomodulation by promoting macrophage polarization from a proinflammatory M1 type to an anti-inflammatory and tissue-healing M2 type [164]. This modulation, mediated predominantly by microRNAs miR-21 and miR-223, results in suppression of proinflammatory cytokines IL-6 and TNF-α, thereby establishing a wound-healing environment for tissue regeneration [165]. MSC-EVs further directly induce proliferation and migration of dermal fibroblasts and keratinocytes leading to skin repair cell types and hence driving wound closure and re-epithelialization. They also assist in reducing the development of scars by regulating expression of collagen subtypes and transforming growth factor (TGF)-β isoforms, namely increasing type III collagen and TGF-β3 but inhibiting type I collagen and TGF-β1, the combined effect of which is antifibrotic and anti-scar tissue formation [166]. EVs also interact with key pathways such as Wnt that orchestrate wound healing and tissue remodeling. In addition, MSC-EVs play a key role in promoting angiogenesis and extracellular matrix remodeling, which are essential events for healing the structural and functional integrity of the skin [167]. In preclinical in vivo models, particularly murine models, the therapeutic efficacy of locally administered MSC-EVs has consistently demonstrated the capacity to accelerate wound healing processes, including enhanced re-epithelialization, reduced scar width, and improved overall skin architecture [168]. Cumulatively, the combined therapeutic actions of MSCs and their EVs in SSTIs are multifaceted. MSCs act directly to improve tissue healing and immune response, while MSC-EVs provide a cell-free, yet effective, modality that heals by activating many molecular and cellular mechanisms. These findings identify the potential of MSC-EVs as an emerging treatment modality for wound infection control and patient outcome improvement in SSTIs, which necessitates further translational studies and clinical trials.

Dendritic-cell-released EVs (DC-EVs) have complementary functions during soft and dermal tissue infections. Although traditionally recognized for their role in antigen presentation and immune surveillance, DC-EVs are now appreciated for their contribution to tissue repair processes, especially in the context of skin and soft tissue injuries [169,170]. It is likely that the most significant contribution of DC-EVs to wound healing involves the recruitment of MSCs to the wound site. DC-EVs naturally encapsulate and carry chemoattractant molecules such as osteopontin (OPN) and matrix metalloproteinase-9 (MMP-9). Such molecules create a chemotactic gradient that signals MSCs to migrate to the site of the wound [171]. MSCs are very important in wound healing because of their immunomodulatory capacity, secretion of proregenerative factors, and ability to enhance angiogenesis and tissue remodeling. Significantly, the effect of DC-EVs on MSC migration is dose-dependent, suggesting a tightly regulated mechanism by which increasing doses of EVs can increasingly enhance MSC recruitment. While the DC-EVs themselves appear not to directly influence MSC proliferation or differentiation, their ability to direct MSC homing toward damaged tissue is a crucial stepping stone for coordination of the initial stages of wound repair [172]. While the primary evidence is in their chemoattractant function, DC-EVs are also anticipated to play roles in controlling the immune microenvironment of the injury. Immune modulators such as DCs can produce EVs containing anti-inflammatory mediators or propolarizing mediators that provoke macrophage polarization towards the M2 phenotype, with a function in the resolution of inflammation and tissue repair. Although more direct studies of DC-EVs here are required, it is possible to draw analogies from EVs of other immune cells, which are known to influence macrophage function and wound healing [173].

B cell-, T cell-, and macrophage-derived EVs play a significant role in immune modulation, inflammation, and repair of tissue. B cell-EVs are filled with MHC-I/II, CD20, and immune-regulatory proteins and can be stimulated by CD40, IL-4, or BCR activation to enhance EV release. These EVs can carry miRNA cargos (miR-155) and induce CD4^+^ T cell apoptosis, though their role in skin diseases such as bullous pemphigoid (BP) and systemic lupus erythematosus (SLE) remains underexplored [174]. T cell-EVs contain TCR, CD3, FasL, and subset-specific miRNAs, which affect DC maturation, NK inhibition, B cell activation, and cytokine secretion; for instance, Treg-EVs suppress Th1 function, while CD8^+^ T cell-EVs induce tumor stroma apoptosis [175]. Macrophage-EVs are of the M1/M2 phenotype and rich in cytokines, Wnt proteins, and cholesterol and control recipient cell function and inflammation. Though they cause inflammation during chronic diseases, macrophage-EVs also promote healing of diabetic wounds by suppressing proinflammatory mediators. Overall, immune cell-derived EVs are emerging as major regulators of immunity and future therapeutic agents in inflammation, cancer, and skin regeneration [176].

Neutrophil EVs play pivotal roles in innate immune reactions, inflammation, and tissue repair [177]. EVs, which are loaded with antimicrobial proteins, granule enzymes, and surface receptors, are increased in sepsis and inflammation and possess pro- and anti-inflammatory functions. They inhibit microbial growth, modulate platelet–neutrophil interaction, and initiate macrophage autophagy for enhanced pathogen elimination. Neutrophil-EVs can exacerbate inflammation by activating endothelial cells, breaking down extracellular matrix, and triggering keratinocytes in psoriasis or exert anti-inflammatory effects by suppressing cytokine production in NK cells, DCs, and macrophages, as seen in gout and cartilage protection [178]. They also regulate endothelial permeability and vascular remodeling: some EV cargos (S100A8/A9, MPO) undermine barrier integrity, while others (annexin 1) preserve it [155,156]. Because of their dual role, neutrophil-EVs are emerging as important regulators in inflammatory skin diseases, which must be investigated further [179].

Endothelial-progenitor-cell-derived (EPC-EVs) are found to be effective wound healing mediators, particularly of diabetic wounds. EPC-EVs induce angiogenesis by promoting enhanced endothelial cell growth, migration, and tube formation, as seen in in vitro and in vivo models [180]. EPC-EVs promote the viability of epidermal cells such as HaCaT keratinocytes by triggering their proliferation, migration, adhesion, and survival even in high-glucose conditions. Mechanistically, EPC-EVs carry microRNAs such as miR-182-5p and miR-221-3p, which regulate tissue repair genes (miR-182-5p) inhibits PPARG for enhanced epidermal cell activity [181]. Moreover, EPC-EVs activate prominent signaling pathways such as Erk1/2 and RAF/ERK, which play a vital role in angiogenesis and tissue repair [182]. EPC-EVs also influence the level of extracellular matrix proteins (fibronectin, MMPs) and inflammatory cytokines (IL-6, IL-8), thereby creating an appropriate microenvironment for wound healing [183]. EPC-EVs have been locally applied in diabetic mouse models to accelerate wound healing, improve collagen alignment, reduce scar formation, and enhance neovascularization. These multifaceted effects render EPC-EVs a cell-free therapeutic candidate for augmenting healing processes in diabetic, chronic, nonhealing wounds [184].

**Figure 3:**
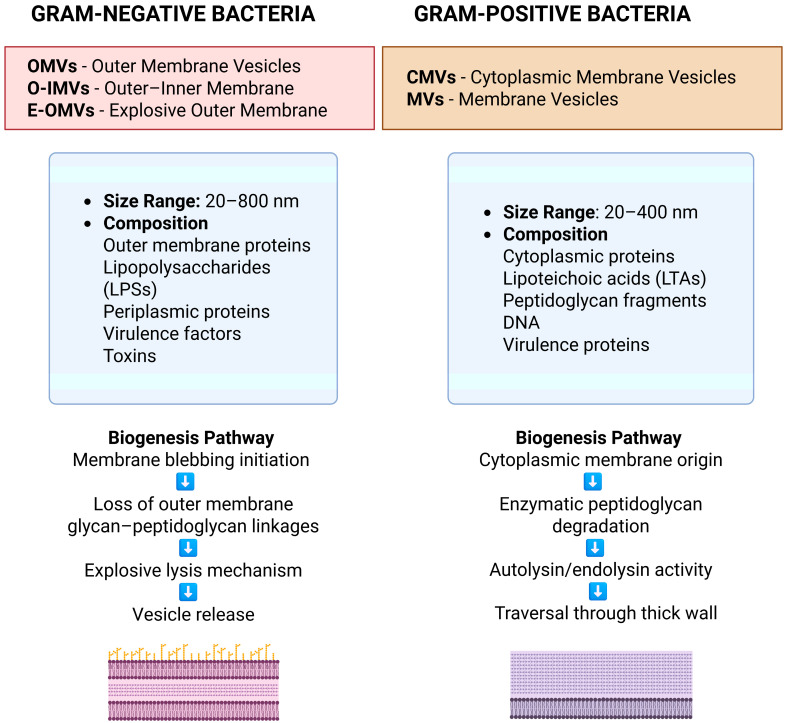
Comparison of EVs from Gram-negative and Gram-positive bacteria.

CMVs from Gram-positive bacteria help regulate soft tissue infections through a variety of mechanisms primarily involving regulation of the host immune response, engaging both innate and adaptive immunity, and facilitating bacterial–host interactions that can affect infection outcomes. CMVs may also stimulate innate immune cells such as macrophages and dendritic cells through pattern recognition receptors such as Toll-like receptor 2 (TLR2). For example, *S. aureus* CMVs activate macrophages via TLR2, leading to NLRP3 inflammasome activation and production of the proinflammatory cytokines IL-1β and IL-18, which are essential for facilitating the innate immune response to infection [185]. CMVs derived from *S. pneumoniae* stimulate immune signaling though NF-κB in macrophages, which increases recruitment of immune cells and inflammatory signaling, contributing to the containment of infection [186]. CMVs derived from *Listeria monocytogenes* can also internalized in nonphagocytic pathways, resulting in autophagic and lysosomal pathways that contribute to the clearance of the pathogen [187]. Meanwhile, CMVs from *Bacillus anthracis* elicit robust IgM responses against toxin components from SSTIs, enhancing survival in infection models [188]. A study on OMVs from the gut commensal *Parabacteroides goldsteinii* (Pg OMVs) demonstrated that orally administered Pg OMVs can reach inflamed skin via the gut–skin axis and significantly reduce skin inflammation in psoriasis-like mouse models [189]. These OMVs suppressed epidermal hyperplasia and inflammatory cell infiltration in skin lesions, effectively ameliorating skin and systemic inflammation. Local subcutaneous delivery of Pg OMVs also yielded similar immunosuppressive effects, suggesting their potential as a therapeutic agent for inflammatory skin diseases. OMVs from commensal gut bacteria such as *Bacteroides fragilis* have been shown to promote the maturation of the immune system and enhance regulatory T cell activation, leading to increased anti-inflammatory cytokine production and prevention of inflammatory disease [190]. A study also explored EVs of *S. aureus*, *Enterococcus* spp., and *Lactobacillus* strains and found them to be protective against HIV-1 infection by virus–cell receptor blocking via steric hindrance and protein receptor shielding. While the study focused on mucosal environments such as the vaginal epithelium, the findings suggest that similar mechanisms may be applicable in skin tissues, where these EVs could contribute to barrier formation and immune modulation. Their ability to inhibit HIV-1 infection underlines a broader immune-protective function, supporting the potential of Gram-positive bacterial EVs as natural immunomodulators in both mucosal and cutaneous contexts [191]. Figure 3 compares the key features of bacterial extracellular vesicles derived from Gram-negative and Gram-positive bacteria.

Although certain strains of *Cutibacterium acnes* (formerly *Propionibacterium acnes*) are already known to be causative of inflammatory acne, recent studies show that EVs of certain phylotypes may have opposite, beneficial effects. Notably, extracellular vesicles of SLST H1 phylotype possess intense anti-inflammatory capacity and intense sebum production inhibition capability on in vitro skin models, as reported by Pol Cros et al. [192], which provides promising leads towards microbiome-directed therapies for acne and associated skin diseases. Likewise, *Staphylococcus epidermidis* EVs, a constituent of the commensal skin microbiota, have demonstrated therapeutic efficacy in AD in re-establishing skin homeostasis. They have immunomodulatory activity by stimulating antimicrobial peptides human β-defensins 2 and 3 and suppress the growth of *S. aureus*, a key accelerator of AD. The vesicles modulate inflammatory gene expression, stimulate cell proliferation and migration, and enhance skin barrier integrity. Together, these behaviors reduce inflammation and induce a healthier and protective skin state [193]. Moreover, EVs from *Lactobacillus plantarum* were reported to enhance anti-inflammatory M2 macrophage polarization, reversing the proinflammatory function of *S. aureus* EVs in AD [194], also bearing witness to the application of such vesicles in treating hyperinflammatory dermatoses such as eczema. Moreover, *S. epidermidis* EVs (str. ATCC12228) inhibited psoriasis-like inflammation through the induction of interleukin-36 receptor antagonist (IL-36Ra), once more affirming the diverse and optimistic roles of bacterial EVs in the modulation of skin inflammation, as reported by Gómez-Chávez et al. (2021) [195].

The medical field recognizes plant-derived EVs as potential therapeutic agents for SSTIs [196]. Biocompatible plant-derived nanovesicles contain different therapeutic components such as proteins and lipids along with RNAs and small metabolites, which demonstrate their therapeutic value. The natural stability and biocompatibility of plant-derived EVs make them valuable for both antimicrobial therapy and wound repair because they can interact with mammalian systems [197]. The antimicrobial nature of plant-derived EVs shows their effectiveness against pathogenic bacteria and fungi. Through the use of mint-leaf-derived nanovesicles in hydrogels (MENV-HG) researchers confirmed their powerful antibacterial effects. The application of these hydrogels on infected skin models produced 99% healing within 10 days while significantly reducing local inflammation [198]. Dandelion-derived vesicle-like nanoparticles (TH-EVNs) block the harmful effects of *S. aureus* exotoxins. The nanoparticles work to repair wounds through their ability to stimulate keratinocyte proliferation and migration which drive essential skin regeneration processes [199]. In addition to antimicrobial activity, plant-derived EVs promote wound healing actively by modulating various phases of tissue regeneration. The regenerative function of PDVs functions through the four classical phases of wound healing: hemostasis, inflammation, proliferation, and remodeling. Grapefruit-derived EVs (GEVs), for instance, are reported to promote keratinocyte proliferation and migration, increase angiogenesis, and decrease oxidative stress, all essential for efficient wound repair [200]. A novel study showed that exosomes from *Flos sophorae immaturus* encapsulated in hydrogel improved spinal cord injury recovery by reducing inflammation and oxidative stress [201]. EVs purified from *Opuntia ficus-indica* fruit (OFI-EVs) may also play a therapeutic role. OFI-EVs were not toxic, and they could reduce oxidative stress and inflammation in vitro. They inhibited proinflammatory cytokines (IL-6, IL-8, TNF-α) in LPS-stimulated THP-1 cells and stimulated the migration of fibroblasts, which reflected their activity in wound healing and made their use in treating chronic skin wounds valid [202]. Carrot-derived EVs were also found to induce IL-10 and Nrf2 activation to inhibit inflammation [203]; similarly, lemon-derived nanovesicles reduced ROS levels induced by H_2_O_2_ and UVB exposure by activating the AhR/Nrf2 pathway in dermal fibroblast cells [204].

Animal-derived EVs are a promising class of bioactive nanocarriers for the improvement of wound healing. Bovine colostrum, the highly nutritious early milk secreted following parturition, is a potent source of regenerative factors, and its EVs have also been found to be very useful in tissue repair [205]. Kim et al. (2021) showed that colostrum-derived EVs (Colos EVs) cause dermal fibroblast proliferation, endothelial tube formation, and keratinocyte migration, which are important processes involved in wound re-epithelialization and neovascularization. These EVs are loaded with growth factors and anti-inflammatory cytokines that provide support for healing from the inflammatory phase to the proliferative phase [206]. Bovine milk is another rich and cheap source of EVs with regenerative and immunomodulatory capacity. These vesicles carry high concentrations of microRNAs (e.g., miR-148a, miR-21), growth factors (e.g., TGF-β and VEGF), and bioactive lipids that facilitate keratinocyte migration and ECM remodeling [207,208]. Milk-derived EVs were shown to suppress overinflammation and stimulate dermal cell proliferation and migration, leading to enhanced restoration of the epithelial barrier and decreased scar tissue formation. Their biocompatibility and nonimmunogenic nature also qualify them for use as topically applied therapeutics in the management of acute and chronic wounds [208]. Platelet-derived EVs are a subset of EVs released during platelet activation and aggregation. These vesicles contain a library of growth factors, including PDGF, TGF-β, VEGF, and chemokines, which are produced by platelets, serving important roles in hemostasis and tissue repair [209,210]. Esmaeilzadeh et al. (2024) demonstrated that PEVs can stimulate fibroblast proliferation, enhance angiogenesis, and modulate immune cell recruitment, creating a proregenerative microenvironment [211]. Of particular note, in chronic wounds such as diabetic ulcers, PEVs inhibited local inflammation and supported increased granulation tissue formation and re-epithelialization. With their native hemostatic and regenerative capabilities, PEVs hold significant promise for acute and chronic wound healing.

Overall, sEVs play an important role in communication between cells. They carry microRNAs, proteins, and lipids that can change how our immune system reacts, influencing things such as how our body handles inflammation and wound healing. For instance, they help shift macrophages from a proinflammatory state to a healing state, which is essential for repairing tissues. They also encourage the movement of skin cells and promote the growth of new blood vessels, making them key players in the healing process. Furthermore, because the contents of sEVs reflect the condition of their source cells, they can serve as valuable biomarkers for diagnosis. On the other hand, lEVs, particularly those from platelets and endothelial cells, tend to drive inflammation and promote clotting. They release substances that ramp up inflammation and help trigger the body’s clotting responses during serious infections. lEVs also present antigens and attract other immune cells, which helps track the source of these vesicles. Table 5 organizes the diverse roles of EVs in SSTI management, while Table 6 presents how different types of EVs contribute to SSTI management through immunomodulation, antimicrobial cargo delivery, and tissue repair.

## 6. Clinical Translation and Future Directions

The therapeutic application of EVs, particularly those derived from MSCs, immune cells, and plant sources, offers exciting potential for treating SSTIs. These vesicles exhibit regenerative, immunomodulatory, and antimicrobial properties that could revolutionize the management of both acute and chronic SSTIs. However, clinical translation remains in its infancy, hampered by scientific, technical, and regulatory challenges that must be addressed for broader implementation.

Several early-phase clinical trials (NCT05243368, NCT04652531, NCT04134676) have begun to evaluate the safety and feasibility of EV-based therapies in wound healing, particularly in chronic and diabetic ulcers [212,213]. These studies underscore the promise of EVs in tissue repair, yet widespread clinical adoption is limited because of difficulties in scalable manufacturing, cargo heterogeneity, batch consistency, and delivery optimization [214]. Regulatory bodies emphasize purity, identity, and safety in EV research and use, especially as the discipline heads towards clinical and industrial use. EV purity is important to eliminate contaminants that can cause side effects, and EV identity characterization—what are they and where they originate from—is important considering the broad variety of biological activities of EV subtypes [215,216]. Safety testing is also necessary to make EV preparations free from toxic entities, enabling patient and user safety. Furthermore, for EV-based products to be viable at scale, they must be produced consistently and reproducibly within tightly regulated parameters. To support these ambitions, the International Society for Extracellular Vesicles (ISEV) has released the MISEV (Minimal Information for Studies of Extracellular Vesicles) guidelines that offer a foundation for improving study quality, reproducibility, and adherence to regulatory expectations in EV research [217,218].

To bridge the gap between preclinical research and clinical application, several key priorities must be addressed. First, a deeper mechanistic understanding of EV biodistribution, uptake, and cell-specific interactions is essential. Second, standardized, GMP-compliant production and quality control protocols are needed to ensure reproducibility and safety. Third, engineering approaches that allow for targeted delivery such as surface modification and cargo loading must be optimized to enhance therapeutic precision [219,220].

The integration of nanotechnology-derived delivery systems such as hydrogels, microneedle patches, and nanocomposites with EVs or exosomes, is a promising method for enhancing therapeutic delivery, diagnostics, and tissue engineering. These delivery vehicles exhibit controlled and sustained release, enhanced biocompatibility, and targeted localization of EVs, thereby harnessing the full therapeutic potential. Hydrogels establish a hydrated, biocompatible environment that protects EVs while facilitating dynamic release [221]; microneedle patches create a noninvasive route for transdermal EV administration [222]; and nanocomposites enhance stability, targeting, and responsiveness to stimuli [223]. This integration facilitates uses such as drug delivery to cancer, real-time disease diagnosis, and regenerative medicine in wound healing or tissue regeneration. Future progress can include the development of intelligent, stimulus-responsive delivery systems, personalized EV-based therapeutics with low immunogenicity, and multifunctional theragnostic platforms, positioning EV-based delivery systems at the forefront of next-generation biomedicine [224,225]. In parallel, EV integration with advanced delivery systems such as hydrogels, microneedle patches, or nanocomposites could improve retention at wound sites and allow for controlled, localized release. Safety profiling remains another critical area, particularly regarding immunogenicity and the long-term effects of repeated administration in humans [226].

Looking ahead, well-designed, multicenter randomized controlled trials are crucial to validate EV-based therapies for SSTIs. Furthermore, EVs may evolve into theragnostic tools, enabling both targeted treatment and real-time disease monitoring through biosensors or molecular imaging. This dual functionality could usher in a new era of personalized medicine for infectious and inflammatory skin conditions [227]. As the field advances, interdisciplinary collaboration across cell biology, materials science, clinical medicine, and regulatory affairs will be essential. With strategic research and innovation, EV-based platforms hold the potential to redefine the therapeutic landscape for SSTIs and overcome the escalating challenge of antimicrobial resistance [228].

## 7. Conclusions

SSTIs are becoming a bigger problem, especially with the growing number of pathogens that resist multiple drugs. Treatments currently available are not always effective. This review looks into a promising new option: EVs. These tiny particles could help in several ways, including fighting infections, boosting the immune system, and aiding tissue healing. EVs can come from various sources, such as MSCs, immune cells, bacteria, plants, and even animals. They have various functions, such as helping to change how immune cells work, regulating important substances in the body, breaking down biofilms, and encouraging new blood vessel growth. MSC-derived EVs are particularly interesting because they could be a way to treat tough SSTIs without using live cells. However, translation to clinical practice is still hindered by problems in standardization, delivery, scale-up, and regulatory approval. The path forward must include overcoming these challenges through technological innovation, clinical validation, and regulatory harmonization. EV-based therapies represent a promising adjunct or alternative to conventional antimicrobial therapies and may herald a paradigm shift in SSTI treatment, especially in the era of antimicrobial resistance.

## Figures and Tables

**Table 1:** Summary of pathogens involved in SSTIs.

Pathogen	Common Infections	Key Virulence Factors	Resistance Mechanisms/Notable Strains	Treatment Considerations
***Staphylococcus aureus*** ***(incl. MRSA)***	Impetigo, cellulitis, abscesses, necrotizing fasciitis	Adhesins (ClfA/B, FnBPs), Protein A, α-toxin, biofilm, PVL (CA-MRSA), TSST-1, wall teichoic acid	MRSA (mecA gene, SCCmec, ACME), multidrug resistance	Clindamycin, doxycycline, TMP-SMX, vancomycin; monitor resistance
***Streptococcus pyogenes*** ***(GAS)***	Cellulitis, erysipelas, necrotizing fasciitis, STSS	M protein, streptolysins O/S, SpeA/B/C, streptokinase, DNase, C5a peptidase, hyaluronidase	Limited resistance; strain diversity (Class I: rheumatic fever, Class II: APSGN)	High-dose penicillin + clindamycin; early intervention critical
***Clostridium perfringens***	Gas gangrene (clostridial myonecrosis), deep wound infections	Alpha-toxin (lecithinase), theta-toxin (PFO), spore formation	Rare resistance, but rapid progression increases risk	Surgical debridement + high-dose IV penicillin; consider hyperbaric O_2_
***Pseudomonas aeruginosa***	Burn wound infections, hot tub folliculitis	Biofilm, elastases, exotoxin A, pyocyanin	MDR, efflux pumps, β-lactamases	Piperacillin–tazobactam, ceftazidime, carbapenems; tailored by sensitivity
***Escherichia coli***	SSTIs in contaminated water exposure	Endotoxins, adhesins, invasins (in pathogenic strains)	ESBL-producing strains (esp. O157:H7)	Empirical broad-spectrum antibiotics; adjust per susceptibility
***Aeromonas hydrophila***	Water-exposed cellulitis, necrotizing fasciitis	Hemolysins, aerolysin, proteases	Emerging MDR	Fluoroquinolones or TMP-SMX; debridement if severe
***Pasteurella multocida***	Cat bite infections (rapid onset cellulitis)	Capsule, adhesins, LPS	Beta-lactamase production (occasionally)	Penicillin or amoxicillin–clavulanate; early treatment key
***Capnocytophaga canimorsus***	Dog bite-related systemic infections, sepsis	Sialidase, capsule, immune evasion enzymes	Beta-lactamase; high risk in asplenic hosts	IV penicillin G or β-lactam/β-lactamase inhibitor; urgent care required
***Eikenella corrodens***	Human bite/clenched fist injuries	Lysins, biofilm-forming capacity	Beta-lactamase in some isolates	Amoxicillin–clavulanate; surgical drainage if abscess forms
***Vibrio vulnificus***	Necrotizing fasciitis, wound sepsis (saltwater exposure)	Cytolysin, metalloproteases, capsule	Intrinsic resistance to some beta-lactams	Doxycycline + third-gen cephalosporin; urgent debridement
***Mycobacterium marinum***	Chronic granulomatous infection (“fish tank granuloma”)	Slow growth, granuloma formation	Intrinsic resistance; slow response to therapy	Clarithromycin + rifampin or ethambutol; prolonged therapy

Abbreviations: ClfA/B (Clumping Factor A/B); FnBPs (Fibronectin-Binding Proteins); PVL (Panton-Valentine Leukocidin); CA-MRSA (Community-Associated Methicillin-Resistant Staphylococcus aureus); TSST-1 (Toxic Shock Syndrome Toxin-1); MRSA (Methicillin-Resistant Staphylococcus aureus); SCCmec (Staphylococcal Cassette Chromosome mec); ACME (Arginine Catabolic Mobile Element); TMP-SMX (Trimethoprim–Sulfamethoxazole); SpeA/B/C (Streptococcal Pyrogenic Exotoxins A/B/C); GAS (Group A Streptococcus); STSS (Streptococcal Toxic Shock Syndrome); APSGN (Acute Post-Streptococcal Glomerulonephritis); PFO (Perfringolysin O); MDR (Multidrug-Resistant); SSTI (Skin and Soft Tissue Infection); ESBL (Extended-Spectrum Beta-Lactamase); O157-H7 (Escherichia coli O157:H7); LPS (Lipopolysaccharide).

**Table 2:** Overview of treatment approaches for SSTIs.

SSTI Type	First-Line Antimicrobials	Adjunctive Therapies	Recent Advances
**Purulent (e.g., abscess)**	I&D ± TMP-SMX, doxycycline, or clindamycin	Wound care, hygiene education, follow-up	Long-acting lipoglycopeptides (dalbavancin, oritavancin)
**Nonpurulent cellulitis**	Cephalexin or IV cefazolin; vancomycin if MRSA suspected	Limb elevation, hygiene education	PK/PD-optimized dosing, serum drug monitoring
**Necrotizing fasciitis**	IV vancomycin + cefepime or piperacillin–tazobactam + clindamycin/linezolid	Urgent surgical debridement, IVIG, HBOT	Prolonged/continuous β-lactam infusion, MSCs/EVs
**Animal bites**	Amoxicillin–clavulanate; doxycycline (penicillin allergy)	Wound cleansing, tetanus prophylaxis	Awareness of zoonotic infections, resistance patterns
**Folliculitis**	Topical agents; systemic if extensive	Hygiene education, topical antibiotics	Improved topical agents, resistance surveillance
**Resistant/toxin-producing**	Delafloxacin, omadacycline; linezolid, tedizolid	Toxin suppression (clindamycin, linezolid, tedizolid)	Next-gen oxazolidinones (tedizolid)
**Polymicrobial infections**	Broad-spectrum β-lactam + clindamycin or linezolid	Combination therapy, supportive care	Regenerative strategies (MSCs, EVs)

Abbreviations: SSTI (Skin and Soft Tissue Infection); I&D (Incision and Drainage); TMP-SMX (Trimethoprim–Sulfamethoxazole); IV (Intravenous); MRSA (Methicillin-Resistant Staphylococcus aureus); PK/PD (Pharmacokinetics/Pharmacodynamics); IVIG (Intravenous Immunoglobulin); HBOT (Hyperbaric Oxygen Therapy); MSCs/EVs (Mesenchymal Stem Cells/Extracellular Vesicles).

**Table 3:** Summary of different types of EVs.

Feature	Small EVs (Exosomes)	Large EVs (Microvesicles/Ectosomes)
**Size**	40–150 nm	100–1000 nm (can exceed 1 µm)
**Origin**	Endosomal system (MVB fusion)	Plasma membrane (direct budding)
**Biogenesis**	ESCRT-dependent/independent pathways	Calcium-triggered membrane blebbing and cytoskeletal remodeling
**Key Regulators**	Rab GTPases (RAB27a/b, RAB11), SNAREs	ARF6, Rho GTPases
**Markers**	CD9, CD63, CD81, ALIX, TSG101, HSP70	Annexin A1, ARF6, integrins
**Cargo**	Nucleic acids, tetraspanins, signaling proteins	Ribosomal proteins, RNA biogenesis factors, cytoplasmic contents
**Functions**	Intercellular communication, immune modulation, adhesion	Inflammation, coagulation, metastasis, cell signaling
**Sedimentation (UC)**	~100,000× *g*	~10,000–20,000× *g*

Abbreviations: EVs (Extracellular Vesicles); MVB (Multivesicular Body); ESCRT (Endosomal Sorting Complex Required for Transport); Rab GTPases (Ras-related in brain GTP-binding proteins); RAB27a/b, RAB11 (Rab family members involved in EV trafficking and release); SNARE (Soluble NSF Attachment Protein Receptor); ARF6 (ADP-Ribosylation Factor 6); Rho GTPases (Ras Homolog GTP-binding proteins); CD9/CD63/CD81 (Tetraspanins—EV surface markers); ALIX (ALG-2-Interacting Protein X); TSG101 (Tumor Susceptibility Gene 101 protein); HSP70 (Heat Shock Protein 70); Annexin A1 (Phospholipid-binding protein involved in membrane dynamics); UC (Ultracentrifugation).

**Table 4:** Pathogen-derived EVs in SSTI pathogenesis.

Microorganism	EV Type	Cargo	Pathogenic Roles in SSTIs	References
***Staphylococcus aureus*** **(Gram-positive)**	Cytoplasmic membrane vesicles (CMVs)/SA-EVs	α-Hemolysin, Protein A, β-lactamases, Proteases, DNA	Disrupts epidermal barrier; induces proinflammatory cytokines (IL-6, TNF-α), biofilm formation, immune evasion, antibiotic resistance	[123,124,125,126,127,128,129,130]
***Pseudomonas aeruginosa*** **(Gram-negative)**	Outer membrane vesicles (OMVs)	Quorum-sensing molecules, extracellular matrix proteins	Supports polymicrobial biofilm maturation, modulates host immunity, enhances antibiotic resistance in chronic wounds	[131]
***Helicobacter pylori***, ***Porphyromonas gingivalis***	OMVs	LPS, OmpA, toxins, small RNAs	Disrupts mucin layers and tight junctions, promotes colonization and tissue invasion, induces NF-κB/MAPK pathways and pyroptosis	[134,135,136,137]
***Neisseria gonorrhoeae***	OMVs	PorB protein	Induces macrophage apoptosis, impairs immune clearance	[138]
***Escherichia coli***, ***Shigella flexneri***	OMVs	Adhesion/invasion proteins (Ail, Ipa)	Promotes adhesion, host invasion, and immune modulation	[139,140]
**Fungal: *Malassezia sympodialis***	EVs	Mala s1, nucleic acids, lysophospholipases	Triggers IL-4 and ICAM-1 in keratinocytes, disrupts barrier function via lipid degradation	[143,144]
**Fungal: *Candida albicans***	EVs	Proteins, lipids, RNAs, enzymes	Promotes biofilm formation, drug resistance, cytokine production (IL-6, IL-10, TNF-α), impairs wound healing	[145,146]
**Fungal: *Cryptococcus neoformans***	EVs	Virulence factors	Modulates macrophage response, enhances inflammation and fungal dissemination in cutaneous cryptococcosis	[147]

Abbreviations: (EV (Extracellular Vesicle); CMVs (Cytoplasmic membrane vesicles); SA-EV (Staphylococcus aureus–derived Extracellular Vesicle); SSTi (Skin and Soft Tissue Infection); IL-6 (Interleukin-6); TNF-α (Tumor Necrosis Factor-alpha); OMVs (Outer Membrane Vesicles); LPS (Lipopolysaccharide); OmpA (Outer Membrane Protein A); NF-κB/MAPK (Nuclear Factor-kappa B/Mitogen-Activated Protein Kinase); PorB (Porin B); Ail (Attachment-Invasion Locus protein); Ipa (Invasion Plasmid Antigen); IL-4 (Interleukin-4); ICAM-1 (Intercellular Adhesion Molecule 1); Mala s1 (Malassezia allergen s1); IL-10 (Interleukin-10).

**Table 5:** Role of EVs in SSTI suppression and wound healing.

EV Source	Key Components/Cargo	Primary Mechanisms	Therapeutic Functions	Specific Applications in SSTIs	Clinical Outcomes
Human Cell-Derived EVs					
**Keratinocyte** **-** **EVs**	IL-10, TGF-β, miR-146a, miR-21, β-defensin 2, S100A12, CXCLs, MHC molecules	Immune cell modulation, M1→M2 polarization, T cell proliferation, AMP delivery	Anti-inflammatory, antimicrobial, barrier enhancement, re-epithelialization	Antibacterial action against *S. aureus*, keratinocyte proliferation/migration, ECM remodeling	Reduced inflammation, enhanced barrier, accelerated healing, chronic inflammation prevention
**Fibroblast** **-** **EVs**	Cathepsin B, MMP-1, TGF-binding proteins, collagen-related miRNAs	ECM remodeling, keratinocyte migration, oxidative stress protection	Tissue repair, antioxidant effects, cellular migration support	Wound healing, matrix remodeling in infected tissue, PAMP/cytokine response	Enhanced regeneration, inflammatory regulation, adaptive wound healing
**MSC-EVs**	miR-21, miR-223, Type III collagen, TGF-β3, Wnt components	M1→M2 polarization, cell proliferation, anti-fibrosis, angiogenesis	Immunomodulation, tissue regeneration, anti-scarring	Wound closure, re-epithelialization, scar reduction	IL-6/TNF-α suppression, improved skin architecture, reduced scar width
**Adipocyte-EVs**	IL-10, MMPs, collagen regulators	Proinflammatory suppression, ECM remodeling, vascularization support	Inflammation control, tissue protection, barrier restoration	Chronic wound therapy, diabetic wounds, infection resistance	Reduced tissue damage, improved healing, barrier function restoration
**DC-EVs**	Osteopontin, MMP-9, anti-inflammatory mediators	MSC recruitment, immune microenvironment regulation, M2 polarization	Cellular recruitment, tissue repair coordination, immune regulation	MSC homing, immune surveillance, wound repair processes	Enhanced MSC recruitment, coordinated healing, regulated immune response
**Neutrophil** **-EVs**	Antimicrobial proteins, granule enzymes, S100A8/A9, MPO, Annexin 1	Microbial inhibition, platelet interaction, macrophage autophagy, endothelial regulation	Antimicrobial action, inflammation modulation, vascular regulation	Pathogen elimination, skin barrier maintenance, psoriasis/eczema therapy	Enhanced clearance, dual inflammatory effects, preserved barrier
**EPC-EVs**	miR-182-5p, miR-221-3p, fibronectin, MMPs, IL-6, IL-8	Endothelial migration, tube formation, ERK1/2 & RAF/ERK activation	Angiogenesis, tissue repair, diabetic wound healing	Keratinocyte activation, neovascularization, chronic wound treatment	Accelerated healing, improved collagen alignment, reduced scarring
**Bacterial EVs**					
**Gram-positive CMVs**	TLR2 ligands, IL-1β, IL-18, NF-κB activators, IgM-inducing components	TLR2/NLRP3 activation, NF-κB signaling, autophagy/lysosome pathways	Innate immunity, adaptive immunity, immune cell recruitment	Immune signaling (e.g., *S. aureus*, *S. pneumoniae*), pathogen elimination (*L. monocytogenes*, *B. anthracis*)	Enhanced immune response, pathogen clearance, improved survival rates
**Commensal Bacterial EVs**	Anti-inflammatory factors, Treg activators, β-defensins 2 and 3	Regulatory T cell activation, cytokine production, pathogen inhibition	Skin homeostasis, microbiome balance, inflammation suppression	*Pg* OMVs for psoriasis, *S. epidermidis* in AD, *C. acnes* in acne, HIV protection	Restored skin integrity, reduced inflammation, microbiome improvement
**Plant-Derived EVs**	Proteins, lipids, RNAs, metabolites, IL-10 inducers, Nrf2 activators	Antimicrobial action, keratinocyte activation, angiogenesis, oxidative stress modulation	Wound healing, antioxidant protection, antimicrobial therapy	Mint (MENV-HG), dandelion (TH-EVNs), grapefruit (GEVs), carrot/lemon antioxidants	~99% healing in 10 days, reduced inflammation, enhanced regeneration
**Animal-Derived EVs**					
**Bovine Colostrum EVs**	Growth factors, anti-inflammatory cytokines, miR-148a, miR-21, TGF-β, VEGF	Fibroblast proliferation, angiogenesis, ECM remodeling	Neovascularization, re-epithelialization, anti-inflammatory support	Acute/chronic wound repair, inflammatory to proliferative phase transition	Improved vascularization, reduced inflammation, faster healing
**Platelet EVs**	PDGF, VEGF, TGF-β, chemokines, hemostatic factors	Fibroblast proliferation, immune cell recruitment, angiogenesis, hemostasis	Immune modulation, tissue regeneration, granulation tissue support	Diabetic ulcer therapy, chronic wound healing, re-epithelialization	Accelerated healing, enhanced granulation tissue, inflammation resolution

Abbreviations: (EVs (Extracellular Vesicles); IL-10 (Interleukin-10); TGF-β (Transforming Growth Factor-beta); miR-146a/miR-21/miR-223/miR-155/miR-182-5p/miR-221-3p (microRNAs); M1/M2 (Macrophage phenotypes); S100A12/S100A8/9 (S100 Calcium-Binding Proteins); CXCL1/3/5/6 (C-X-C Motif Chemokine Ligands); MMP-1/MMP-9 (Matrix Metalloproteinases); IL-1β/IL-6/IL-8/IL-36Ra (Interleukins); TNF-α (Tumor Necrosis Factor-alpha); ECM (Extracellular Matrix); MSCs (Mesenchymal Stem Cells); OPN (Osteopontin); MHC-I/II (Major Histocompatibility Complex Class I/II); CD4/CD3 (Cluster of Differentiation); TCR (T Cell Receptor); FasL (Fas Ligand); Th1 (T helper type 1 cells); DCs (Dendritic Cells); NK (Natural Killer cells); Wnt (Wingless/Integrated pathway); MPO (Myeloperoxidase); TLR2 (Toll-Like Receptor 2); NF-κB (Nuclear Factor-kappa B); NLRP3 (NOD-, LRR- and pyrin domain-containing protein 3); HIV (Human Immunodeficiency Virus); Nrf2 (Nuclear factor erythroid 2–related factor 2) (→: lead to).

**Table 6:** EV types and their therapeutic mechanisms in SSTIs.

EV Source	Immunomodulation	Antimicrobial Cargo Delivery	Tissue Repair
**Keratinocyte-Derived EVs**	• IL-10, TGF-β, miR-146a, miR-21 • Polarize M1→M2 macrophages • Modulate T cells • Suppress excess immune activation	• β-defensin 2, S100A12, human β-defensin 3 • CXCL1/3/5/6 chemoattractants • Activity against *S. aureus*	• Promote proliferation, migration, re-epithelialization • Enhance barrier function • Activate fibroblasts via cathepsin B, MMP-1
**Fibroblast-Derived EVs**	• Modulated by infection and cytokines • Resolve or exacerbate inflammation	• Modified cargo during infection • Influence immune cell recruitment	• Enhance keratinocyte migration • Protect from oxidative stress • Support matrix remodeling and wound healing
**Adipocyte-Derived EVs**	• Suppress IL-1β, IL-6, TNF-α • Promote IL-10 • Reduce excessive inflammation	• Indirect antimicrobial via immune control	• Improve vascularization and barrier function • Stimulate ECM remodeling, MMPs, and collagen deposition
**MSC-Derived EVs**	• Polarize M1→M2 via miR-21, miR-223 • Suppress IL-6, TNF-α • Create prohealing immune milieu	• Indirect antimicrobial effects • Enhance immune clearance	• Stimulate fibroblast and keratinocyte proliferation • Promote angiogenesis • Regulate collagen subtypes (↑III, ↓I), reduce scarring
**Dendritic-Cell-Derived EVs**	• Polarize macrophages to M2 • Control immune environment	• Support immune surveillance and pathogen detection	• Recruit MSCs via OPN, MMP-9 • Establish chemotactic gradients • Initiate coordinated wound repair
**B Cell-Derived EVs**	• Contain MHC-I/II, miR-155 • Induce CD4+ T cell apoptosis • Modulate adaptive immunity	• Enhance pathogen recognition	• Support immune-mediated tissue repair
**T Cell-Derived EVs**	• Contain TCR, CD3, FasL, miRNAs • Treg-EVs suppress Th1 • Modulate DCs, NK cells	• Stimulate immune cell activation	• Influence tissue remodeling via cytokine modulation
**Macrophage-Derived EVs**	• Rich in cytokines, Wnt proteins • M1/M2 phenotype specific • Control recipient immune response	• Enhance phagocytic and antimicrobial responses	• Support tissue regeneration • Promote diabetic wound healing
**Neutrophil-Derived EVs**	• Dual pro-/anti-inflammatory effects • Suppress cytokines in NKs, DCs • Modulate endothelial activation	• Contain MPO, S100A8/9, enzymes • Inhibit microbial growth	• Modulate vascular permeability • Carry annexin-1 • Support tissue remodeling
**Endothelial Progenitor Cell EVs**	• Modulate IL-6, IL-8 • Create balanced immune milieu	• Indirect support via improved barrier	• Promote angiogenesis, keratinocyte proliferation • miR-182-5p, miR-221-3p regulate repair genes
**Gram^+^ Bacterial EVs (CMVs)**	• Activate macrophages via TLR2 • Trigger NF-κB, NLRP3 • Enhance Treg function	• Direct antimicrobial effects • Block viral receptors (e.g., HIV) • Microbiome stabilization	• Promote immune maturation • Enhance barrier and tissue homeostasis
**Commensal Bacterial EVs**	• *S. epidermidis* induces β-defensins • IL-36Ra induction • *L. plantarum* promotes M2 polarization	• Suppress *S. aureus* growth • Enhance AMP production	• Reduce inflammation • Promote skin integrity and cell migration
**Plant-Derived EVs**	• Suppress IL-6, IL-8, TNF-α • Induce IL-10 • Activate Nrf2	• Antibacterial (mint, dandelion EVs) • Inhibit *S. aureus* exotoxins • Antifungal activity	• Stimulate angiogenesis • Promote re-epithelialization • Reduce oxidative stress
**Animal-Derived EVs**	• Bovine colostrum: TGF-β, anti-inflammatory factors • Platelet EVs: recruit immune cells	• Nonimmunogenic delivery • Indirect pathogen clearance	• Promote fibroblast proliferation • ECM remodeling • Re-epithelialization • Granulation tissue formation

Abbreviations: (EVs (Extracellular Vesicles); IL-10 (Interleukin-10); TGF-β (Transforming Growth Factor-beta); miR-146a/miR-21/miR-223/miR-155/miR-182-5p/miR-221-3p (microRNAs); M1/M2 (Macrophage Phenotypes); S100A12/S100A8/9 (S100 Calcium-Binding Proteins); CXCL1/3/5/6 (C-X-C Motif Chemokine Ligands); MMP-1/MMP-9 (Matrix Metalloproteinases); IL-1β/IL-6/IL-8/IL-36Ra (Interleukins); TNF-α (Tumor Necrosis Factor-alpha); ECM (Extracellular Matrix); MSCs (Mesenchymal Stem Cells); OPN (Osteopontin); MHC-I/II (Major Histocompatibility Complex Class I/II); CD4/CD3 (Cluster of Differentiation); TCR (T Cell Receptor); FasL (Fas Ligand); Th1 (T Helper Type 1 Cells); DCs (Dendritic Cells); NK (Natural Killer Cells); Wnt (Wingless/Integrated Pathway); MPO (Myeloperoxidase); TLR2 (Toll-Like Receptor 2); NF-κB (Nuclear Factor-kappa B); NLRP3 (NOD-, LRR- and Pyrin Domain-Containing Protein 3); HIV (Human Immunodeficiency Virus); Nrf2 (Nuclear Factor Erythroid 2–Related Factor 2). ((↑: upregulated ↓downregulated).
